# *Para*-Halogenation Affects Monoamine Transporter Inhibition Properties and Hepatocellular Toxicity of Amphetamines and Methcathinones

**DOI:** 10.3389/fphar.2019.00438

**Published:** 2019-04-24

**Authors:** Dino Luethi, Melanie Walter, Xun Zhou, Deborah Rudin, Stephan Krähenbühl, Matthias E. Liechti

**Affiliations:** ^1^ Division of Clinical Pharmacology and Toxicology, Department of Biomedicine, University Hospital Basel, University of Basel, Basel, Switzerland; ^2^ Swiss Centre for Applied Human Toxicology (SCAHT), University of Basel, Basel, Switzerland

**Keywords:** amphetamine, cathinone, liver injury, mitochondria, monoamine, transporter

## Abstract

Halogenated derivatives of amphetamine-type stimulants are appearing on the drug market, often with altered pharmacological profile and sometimes different legal status compared to the non-halogenated substances. The aim of the present study was to investigate the pharmacological profile and hepatocellular toxicity of *para*-halogenated amphetamines and cathinones. The potential of amphetamine, 4-fluoroamphetamine, 4-chloroamphetamine, methcathinone, 4-fluoromethcathinone, and 4-chloromethcathinone to inhibit the monoamine transporters for norepinephrine, dopamine, and serotonin was determined in transporter-transfected human embryonic kidney 293 cells. Cell membrane integrity, ATP content, oxygen consumption rate, and superoxide levels were measured in human hepatoma HepG2 cells after exposure to the substances for 24 h. All compounds inhibited the norepinephrine transporter at submicromolar concentrations and the dopamine transporter at low micromolar concentrations. The selectivity of the compounds to inhibit the dopamine *versus* serotonin transporter decreased with increasing size of the *para*-substituent, resulting in potent serotonin uptake inhibition for the halogenated derivatives. All substances depleted the cellular ATP content at lower concentrations (0.25–2 mM) than cell membrane integrity loss occurred (≥0.5 mM), suggesting mitochondrial toxicity. The amphetamines and 4-chloromethcathinone additionally impaired the mitochondrial respiratory chain, confirming mitochondrial toxicity. The following toxicity rank order for the *para*-substituents was observed: chloride > fluoride > hydrogen. In conclusion, *para*-halogenation of stimulants increases the risk for serotonergic neurotoxicity. Furthermore, *para*-halogenation may increase hepatic toxicity mediated by mitochondrial impairment in susceptible users.

## Introduction

**Figure 1:**
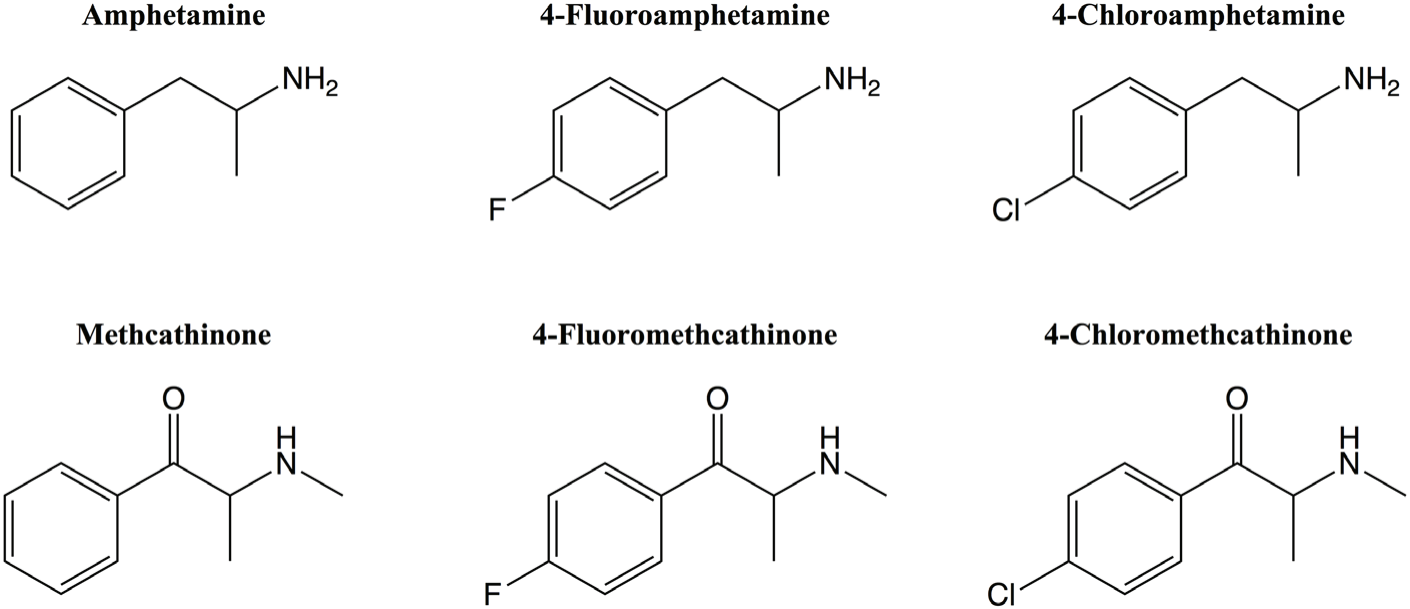
*Para*-halogenated amphetamine and methcathinone derivatives.

Halogenation of illicit amphetamine-type substances represents a tool to create novel designer drugs in clandestine chemistry. Besides having an altered pharmacological profile (Rickli et al., 2015; Mayer et al., 2018; Luethi et al., 2018a), halogenated derivatives may emerge as uncontrolled substances, facilitating their distribution over the Internet. However, halogenation can also result in increased toxicity. For instance, the *para*-chlorinated derivative of amphetamine (4-chloroamphetamine) exerts an increased serotonergic toxicity when compared to the parent compound (Miller et al., 1986; Fuller, 1992; Colado et al., 1993). Other *para*-halogenated stimulants (Figure 1), such as 4-fluoroamphetamine (4-FA), 4-fluoromethcathinone (flephedrone), and 4-chloromethcathinone (clephedrone), have recently appeared on the recreational drug market (Brandt et al., 2010; Taschwer et al., 2014; Linsen et al., 2015; Odoardi et al., 2016; Grifell et al., 2017; Tomczak et al., 2018), but their pharmacological properties and toxicity are currently not well investigated.

Considering the lack of knowledge about the pharmacological and toxicological properties of the halogenated amphetamine and methcathinone (β-keto *N*-methylamphetamine) derivatives, the current study had two principle aims. First, we wanted to determine the effect of *para*-halogenation of amphetamine and methcathinone on the stimulant-type pharmacological potency of these compounds. For that, we obtained monoamine uptake inhibition profiles in monoamine transporter-transfected human embryonic kidney (HEK) 293 cells, which represent a widely used *in vitro* model to study transporter inhibition of stimulants. Second, we investigated the impact of *para*-halogenation of amphetamine and methcathinone on the toxicological profile of these compounds. Since liver injury is a potentially severe complication of amphetamine-type drug intake (Carvalho et al., 2012), we used HepG2 cells for this purpose. HepG2 cells represent a well-established human hepatoma cell line, which has previously been used to study hepatocellular toxicity of stimulants (Dias da Silva et al., 2013, 2015; Luethi et al., 2017). With this study, we aimed on the one hand to assess the hepatotoxic potential of these drugs and on the other hand to put this potential into perspective to their stimulant properties, which may put users at risk of sympathomimetic toxicity.

## Materials and Methods

### Test Substances

Amphetamine, 4-fluoroamphetamine, methcathinone, 4-fluoromethcathinone, and 4-chloromethcathinone were purchased from Lipomed (Arlesheim, Switzerland) with HPLC purity of >98.5%. 4-Chloroamphetamine was purchased from Cayman Chemical (Ann Arbor, MI, USA) with a purity of >98%. All drugs were obtained as racemic hydrochloride salts.

### Cell Line and Culture

Cell culture medium and supplements were purchased from Thermo Fisher Scientific (Basel, Switzerland). HEK 293 cells stably transfected with the human monoamine transporter for norepinephrine (hNET), dopamine (hDAT), or serotonin (hSERT; Tatsumi et al., 1997) were cultured in Dulbecco’s Modified Eagle Medium (DMEM, 4.5 g/L glucose) supplemented with 10% non-heat inactivated fetal calf serum (FCS), 2 mM L-glutamine, 1 mM sodium pyruvate, and 1% MEM non-essential amino acids. Geneticin (250 μg/ml) was used as selection antibiotic. HepG2 cells were cultured in DMEM (1 g/L glucose) supplemented with 10% heat inactivated FCS, 10 mM HEPES buffer, 2 mM GlutaMAX, 1% MEM non-essential amino acids, and penicillin-streptomycin (10,000 U/ml corresponding to 10 mg/ml). Both cell lines were cultured at 37°C in a 5% CO_2_ humidified atmosphere.

### Monoamine Transport Inhibition

The monoamine uptake inhibition of the halogenated amphetamine and methcathinone derivatives was assessed at ambient temperature in transporter-transfected HEK 293 cells at concentrations in the range of 1 nM–900 μM as previously described (Luethi et al., 2018b). As summarized, the cells were detached from the cultivation flask and resuspended in Krebs-Ringer Bicarbonate Buffer (Sigma-Aldrich, Buchs, Switzerland) at a density of 3 million cells per ml. The cell suspension was transferred into a round bottom 96-well plate (100 μl cell suspension per well) and incubated with the compounds of interest for 10 min before [^3^H]norepinephrine (13.1 Ci/mmol, PerkinElmer, Schwerzenbach, Switzerland), [^3^H]dopamine (30.0 Ci/mmol, PerkinElmer, Schwerzenbach, Switzerland), or [^3^H]serotonin (80 Ci/mmol, Anawa, Zürich, Switzerland), which was added at a final concentration of 5 nM for another 10 min. The assay mixtures (100 μl per well) were then transferred into 0.5 ml centrifugation tubes, and the cells were separated from the supernatant through a silicone oil phase by centrifugation at 16,550 g for 3 min. The tubes were then snap frozen in liquid nitrogen, and the cell pellets were cut into 6 ml scintillation vials containing 0.5 ml lysis buffer (0.05 M TRIS-HCl, 50 mM NaCl, 5 mM EDTA, and 1% NP-40 in water). After vigorously shaking the vials for 1 h, 3 ml Ultima Gold (PerkinElmer, Schwerzenbach, Switzerland) was added, and the radioactivity was quantified by liquid scintillation counting using a Packard Tri-Carb Liquid Scintillation Counter 1900 TR. The uptake in the presence of transporter-specific inhibitors (10 μM nisoxetine for NET, 10 μM mazindol for DAT, and 10 μM fluoxetine for SERT) was assessed in order to determine non-specific uptake.

### Cell Membrane Integrity

Cell membrane integrity loss was assessed with the ToxiLight BioAssay Kit (Lonza, Basel, Switzerland) as a marker of cytotoxicity. Briefly, HepG2 cells were seeded in a 96-well plate at a density of 25,000 cells per well. The following day, the cells were treated with 100 μl of the compounds of interest dissolved in medium (0.25, 0.5, 1.0, and 2.0 mM of each drug, and additionally, 0.1 mM of 4-fluoroamphetamine and 4-chloroamphetamine). Triton X-100 (0.5%) was chosen as positive control to induce cell lysis. After 24 h, 50 μl of the ToxiLight assay buffer was added to 20 μl of the supernatant of the drug treatments. After 5 min incubation under shaking, luminescence was measured with a M200 Pro Infinity plate reader (Tecan, Männedorf, Switzerland) and compared to medium control. The amount of viable cells (no cell membrane integrity loss) was calculated in relation to untreated cells and cells lysed with Triton X-100, which were determined to represent 100% and 0% viable cells, respectively.

### ATP Content

The ATP content was assessed with the CellTiter-Glo Luminescent Cell Viability Assay (Promega, Dübendorf, Switzerland). Briefly, HepG2 cells were seeded in a 96-well plate at a density of 25,000 cells per well. The following day, the cells were treated with 100 μl of the test substances dissolved in medium (0.25, 0.5, 1.0, and 2.0 mM of each drug, and additionally, 0.1 mM of 4-fluoroamphetamine and 4-chloroamphetamine). Triton X-100 (0.5%) was chosen as positive control to induce cell lysis. After 24 h incubation, 50 μl of the supernatant was discarded, and 50 μl of CellTiter-Glo reagent was added to each well. The plate was then shaken for 15 min at room temperature to induce cell lysis. Thereafter, luminescence was measured with a M200 Pro Infinity plate reader (Tecan, Männedorf, Switzerland) and compared to medium control.

### Oxygen Consumption

The cellular oxygen consumption rate (OCR) was measured with a Seahorse XF24 Analyzer (Seahorse Biosciences, North Billerica, MA, USA). Briefly, HepG2 cells were seeded in a poly-D-lysine coated Seahorse XF24 cell culture microplate at a density of 100,000 cells per well. The following day, the cells were treated with the compounds of interest dissolved in 100 μl culture medium. After 24 h incubation, the medium was removed, and the cells were washed twice with pre-warmed and unbuffered DMEM (4 mM l-glutamate, 1 mM pyruvate, 1 g/L glucose, 63.3 mM sodium chloride, pH 7.4). Thereafter, 750 μl of unbuffered DMEM was added to each well, and the plate was incubated in a CO_2_ free incubator for 40 min at 37°C. The ports of the XF24 assay cartridge were then loaded with oligomycin, trifluoromethoxy carbonylcyanide phenylhydrazone (FCCP), or rotenone at a final assay concentration of 1 μM, and the cartridge was loaded into the Seahorse XF24 Analyzer. First, oligomycin was injected to inhibit mitochondrial phosphorylation, allowing a quantification of proton leak. Second, oxidative phosphorylation was uncoupled from ATP synthesis by FCCP to determine the maximal respiratory capacity of the cells. Third, rotenone was injected to inhibit complex I of the electron transport chain in order to assess for extramitochondrial respiration, which was then subtracted from basal, leak, and maximal respiration. The protein content was determined using sulforhodamine B staining. The cells were fixed with 100 μl trichloroacetic acid (50% [w/v]), added to each well of the assay plate. After 1 h at 4°C, the cells were washed with deionized water and stained with sulforhodamine B (0.4% [w/v], in 1% [v/v] acetic acid). After 20 min, the cells were rapidly washed with 1% (v/v) acetic acid, and the incorporated dye was solubilized with 100 μl of 10 mM TRIS base. The absorbance was then measured at 490 nm.

### Oxidative Stress

Reactive oxygen species (ROS) production was determined with the mitochondrial superoxide (O_2_^−^) indicator MitoSOX (Thermo Fisher Scientific, Basel, Switzerland), which is a live-cell permeant fluorogenic dye that targets the mitochondria and exhibits red fluorescence upon oxidation by superoxide. Briefly, HepG2 cells were seeded in a 96-well plate at a density of 25,000 cells per well. The following day, the cells were treated with 100 μl of the test substances dissolved in medium (0.25, 0.5, 1.0, and 2.0 mM of each drug, and additionally, 0.1 mM of 4-fluoroamphetamine and 4-chloroamphetamine). Amiodarone (50 μM) was included as positive control. After 24 h incubation, 100 μl of MitoSOX reagent (2.5 μM) was added to each well for 10 min at 37°C protected from light. After that, fluorescence was measured at 510/580 nm with a M200 Pro Infinity plate reader (Tecan, Männedorf, Switzerland). The protein content was assessed with the Pierce BCA Protein Assay Kit (Thermo Fisher Scientific, Basel, Switzerland).

### Mechanisms of Cell Death

Annexin V, a Ca^2+^-dependent phospholipid-binding protein that has a high affinity for phosphatidylserine, was used to determine the percentage of apoptotic cells. Phosphatidylserine is located in the inner cytoplasmic surface of intact cell membranes; in apoptotic cells, phosphatidylserine is translocated to the outer leaflet of the plasma membrane where annexin V binds to it. To assess the percentage of dead cells composed of necrotic and late apoptotic cells, propidium iodide, a red fluorescent dye not permeable to live and apoptotic cells but able to stain permeable necrotic cells by binding to the nucleic acid in the cell, was used. Briefly, 100,000 HepG2 cells per well were seeded in a 48-well plate and cultured overnight. The following day, the cells were treated with 250 μl of the compounds of interest dissolved in medium at concentrations selected based on ATP depletion and cell membrane disruption. Doxorubicin (0.5 μM) was included as positive control. After incubation for 24 h, the cells were harvested with 0.05% trypsin and washed with phosphate buffered saline (pH = 7.4). Afterward, 1 μl Alexa Fluor 488 annexin V staining (Molecular Probes, OR, USA) and 1 μl propidium iodide staining (Molecular Probes, OR, USA) were added to the cell suspension at a final concentration of 10 μg/ml. After 15 min incubation in the dark, the cells were analyzed with a CytoFLEX flow cytometer (Beckman Coulter, IN, USA) gating for 10,000 cells. The data were assessed using FlowJo software 10.08 (Tree Star, Ashland, OR, USA).

### Statistics

All statistical analyses were performed using GraphPad Prism 7.0c (GraphPad Software, La Jolla, CA, USA). For toxicology assays, differences between control and test drugs were calculated with ANOVA followed by Dunett’s test. *p* below 0.05 was considered statistically significant. Monoamine uptake data were fit by nonlinear regression to variable slope sigmoidal dose-response curves to assess IC_50_ values.

## Results

### Monoamine Transporter Inhibition

**Figure 2:**
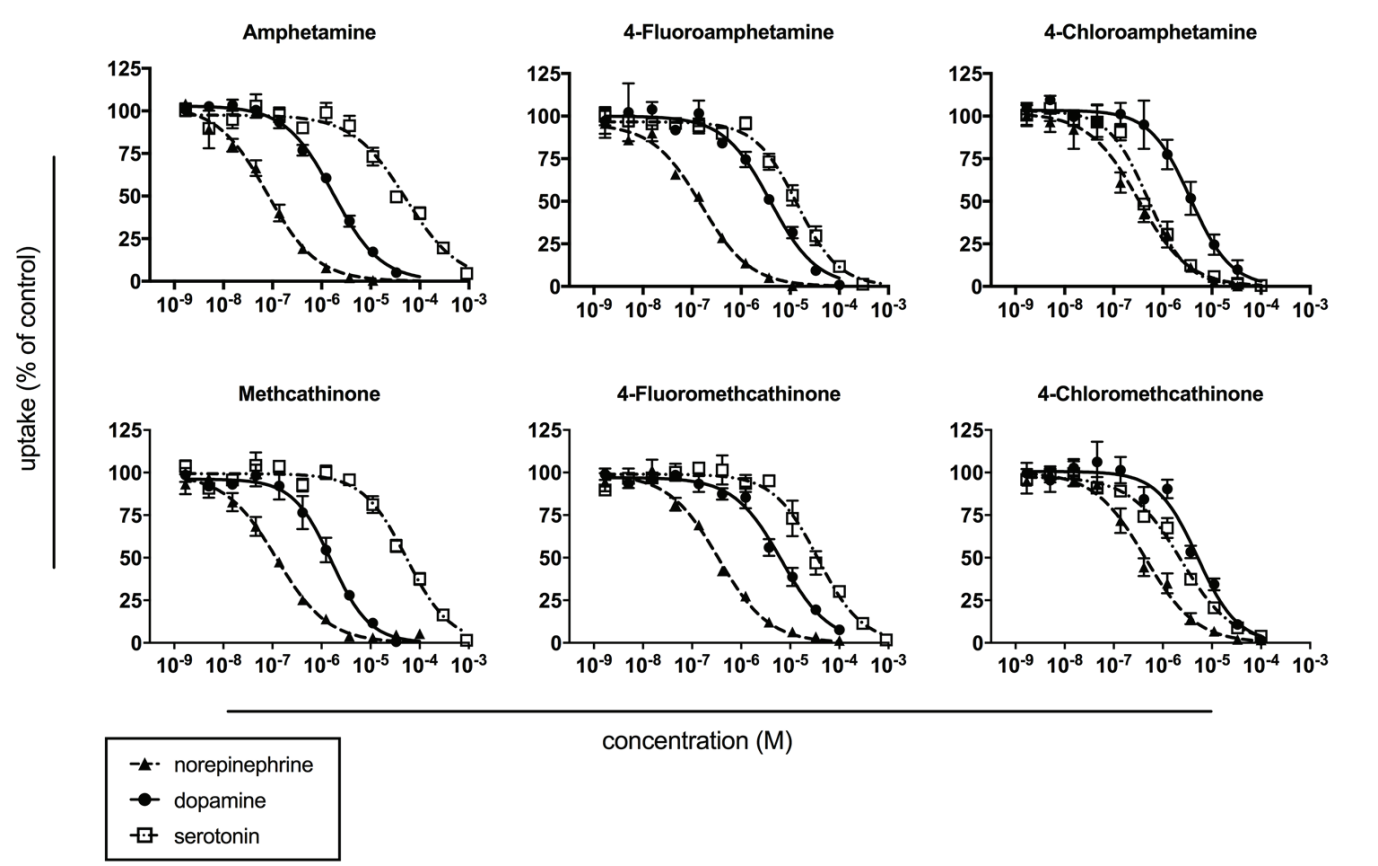
Monoamine uptake inhibition in stably transfected HEK 293 cells that expressed the human NET, DAT, or SERT. Curves were fitted by non-linear regression, and corresponding IC_50_ values are shown in Table 1. The data are presented as the mean ± SEM of three experiments performed in triplicate.

Monoamine uptake inhibition curves are shown in Figure 2, and the corresponding IC_50_ values are listed in Table 1. For all amphetamines and methcathinones, most potent uptake inhibition was observed at NET (IC_50_ values in the range of 0.08–0.43 μM). The compounds displayed similar potential to inhibit dopamine uptake (IC_50_ values in the range of 1.6–6.6 μM) but differed greatly in their potential to inhibit serotonin uptake (IC_50_ values in the range of 0.5–54 μM). *Para*-fluorination increased the inhibition potency at the SERT *versus* DAT roughly one order of magnitude and *para*-chlorination roughly two orders of magnitude compared to the non-substituted amphetamine and methcathinone.

**Table 1:** Monoamine uptake inhibition.

	NET IC_50_ (μM)	DAT IC_50_ (μM)	SERT IC_50_ (μM)	DAT/SERT ratio
Amphetamine	0.078 (0.053–0.113)	1.7 (1.4–2.0)	51 (38–68)	30 (19–49)
4-Fluoroamphetamine	0.15 (0.12–0.21)	3.9 (2.6–5.8)	14 (11–17)	3.6 (1.9–6.5)
4-Chloroamphetamine	0.32 (0.20–0.51)	3.6 (2.6–5.1)	0.49 (0.37–0.65)	0.14 (0.07–0.25)
Methcathinone	0.12 (0.08–0.17)	1.6 (1.2–2.1)	54 (43–68)	34 (20–57)
4-Fluoromethcathinone	0.35 (0.26–0.46)	6.6 (5.2–8.4)	37 (28–49)	5.6 (3.3–9.4)
4-Chloromethcathinone	0.43 (0.30–0.62)	5.1 (3.6–7.2)	2.3 (1.8–3.0)	0.45 (0.25–0.83)

Values are means and 95% confidence intervals (CI). DAT/SERT ratio = 1/DAT IC_50_: 1/SERT IC_50_.

### Cell Membrane Integrity and ATP Content

**Figure 3:**
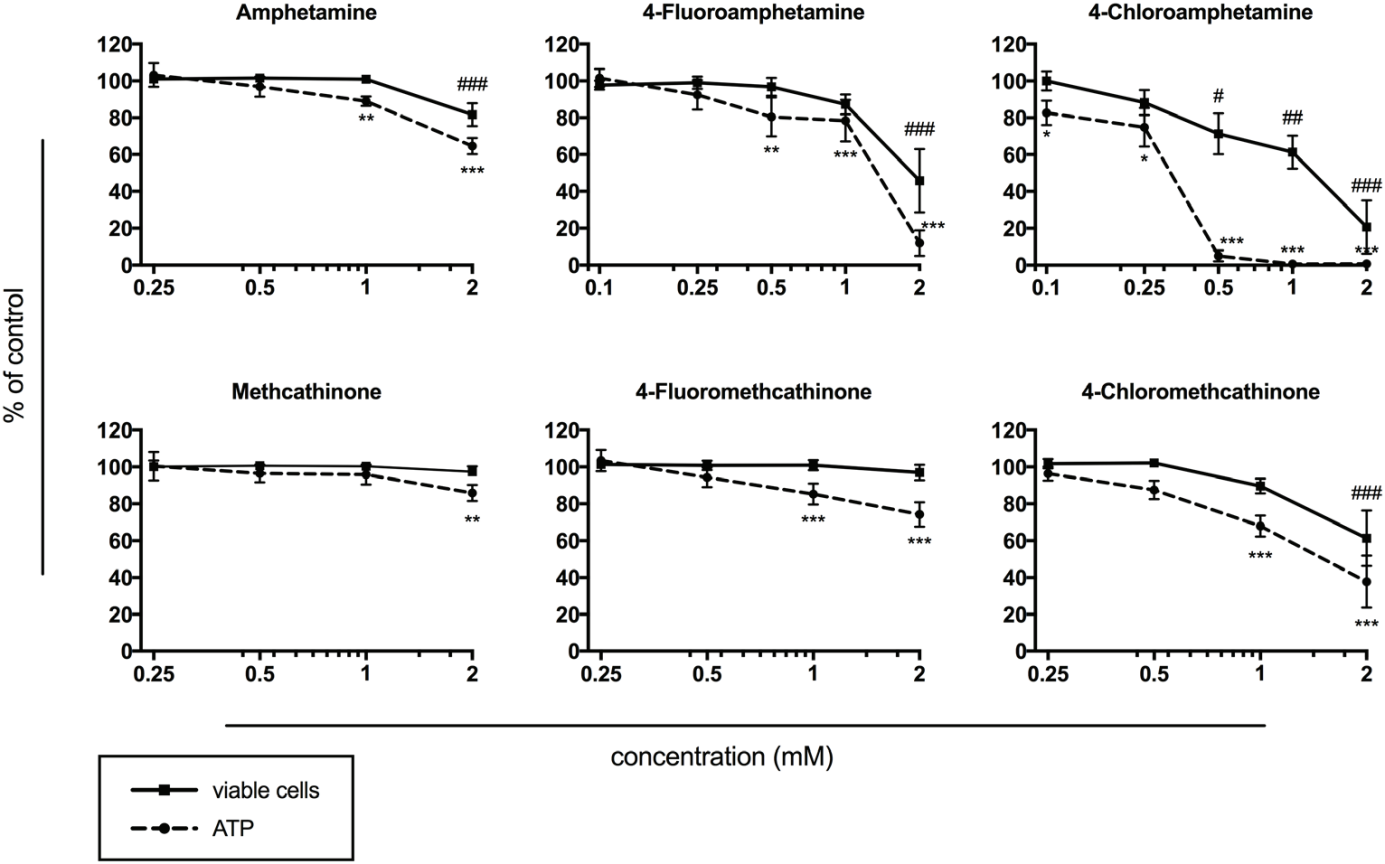
Intracellular ATP content and viable cells expressed by maintained cell membrane integrity after drug exposure of HepG2 cells for 24 h. Data are expressed as mean ± SEM, and numbers in parentheses indicate the number of individual experiments performed in triplicate: amphetamine (4), 4-fluoroamphetamine (7), 4-chloroamphetamine (4), methcathinone (4), 4-fluoromethcathinone (4), and 4-chloromethcathinone (4). Significance levels are given as *p < 0.05, **p < 0.01, ***p < 0.001 (ATP content); #*p* < 0.05, ##*p* < 0.01, ###*p* < 0.001 (viable cells).

In comparison to the interaction with monoamine transporters, the toxicity on HepG2 cells occurred at substantially higher concentrations. A significant and concentration-dependent depletion of ATP was observed at 0.1 mM for 4-chloroamphetamine; at 0.5 mM for 4-fluoroamphetamine; at 1 mM for amphetamine, 4-fluoromethcathinone, and 4-chloromethcathinone; and at 2 mM for methcathinone (Figure 3). 4-Chloroamphetamine disrupted the cell membrane at 0.5 mM, whereas amphetamine, 4-fluoroamphetamine, and 4-chloromethcathinone caused significant cell membrane integrity loss at 2 mM (Figure 3). No cell membrane integrity loss was observed after 24 h exposure of HepG2 cells to methcathinone and 4-fluoromethcathinone at concentrations up to 2 mM.

### Cellular Oxygen Consumption

**Figure 4:**
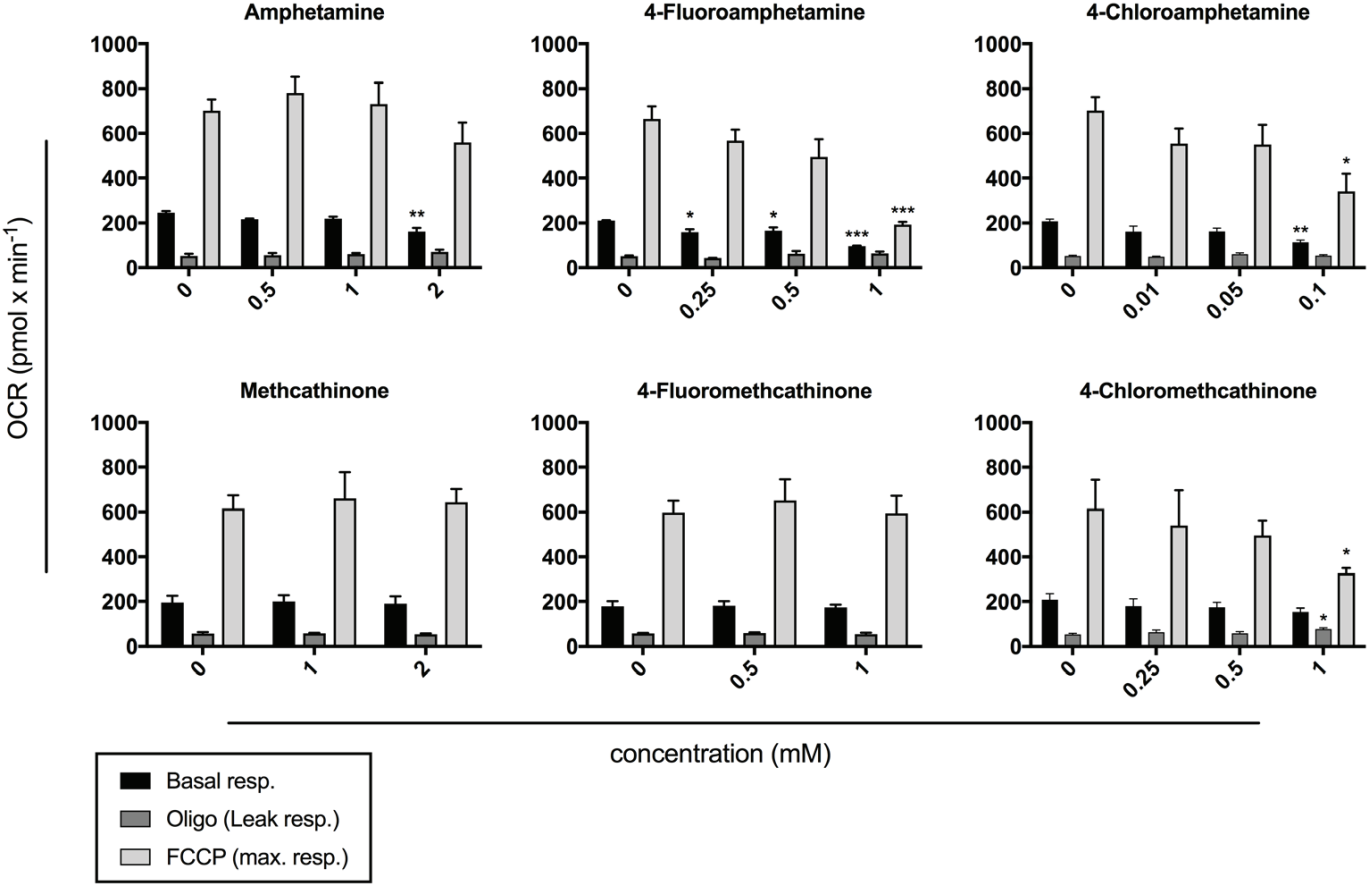
Oxygen consumption rate (OCR) in HepG2 cells after drug exposure for 24 h. Basal respiration, leak respiration, and maximal respiration are expressed as mean ± SEM adjusted to protein content. Numbers in parentheses indicate the number of individual experiments performed in triplicate: amphetamine (3), 4-fluoroamphetamine (3), 4-chloroamphetamine (5), methcathinone (5), 4-fluoromethcathinone (5), and 4-chloromethcathinone (4). Significance levels are given as *p < 0.05, **p < 0.01, ***p < 0.001.

The effect of halogenated amphetamine and methcathinone derivatives on cellular respiration is shown in Figure 4. Amphetamine and its *para*-halogenated derivatives decreased basal respiration in the range of 0.1–2 mM. 4-Fluoroamphetamine and 4-chloroamphetamine additionally decreased the maximal respiratory capacity at 1 and 0.1 mM, respectively. 4-Chloromethcathinone decreased maximal respiration at 1 mM and showed signs of uncoupling of cellular respiration from ATP synthesis at the same concentration. Methcathinone and 4-fluoromethcathinone did not disrupt cellular respiration in the investigated concentration range (≤2 mM).

### Oxidative Stress

**Figure 5:**
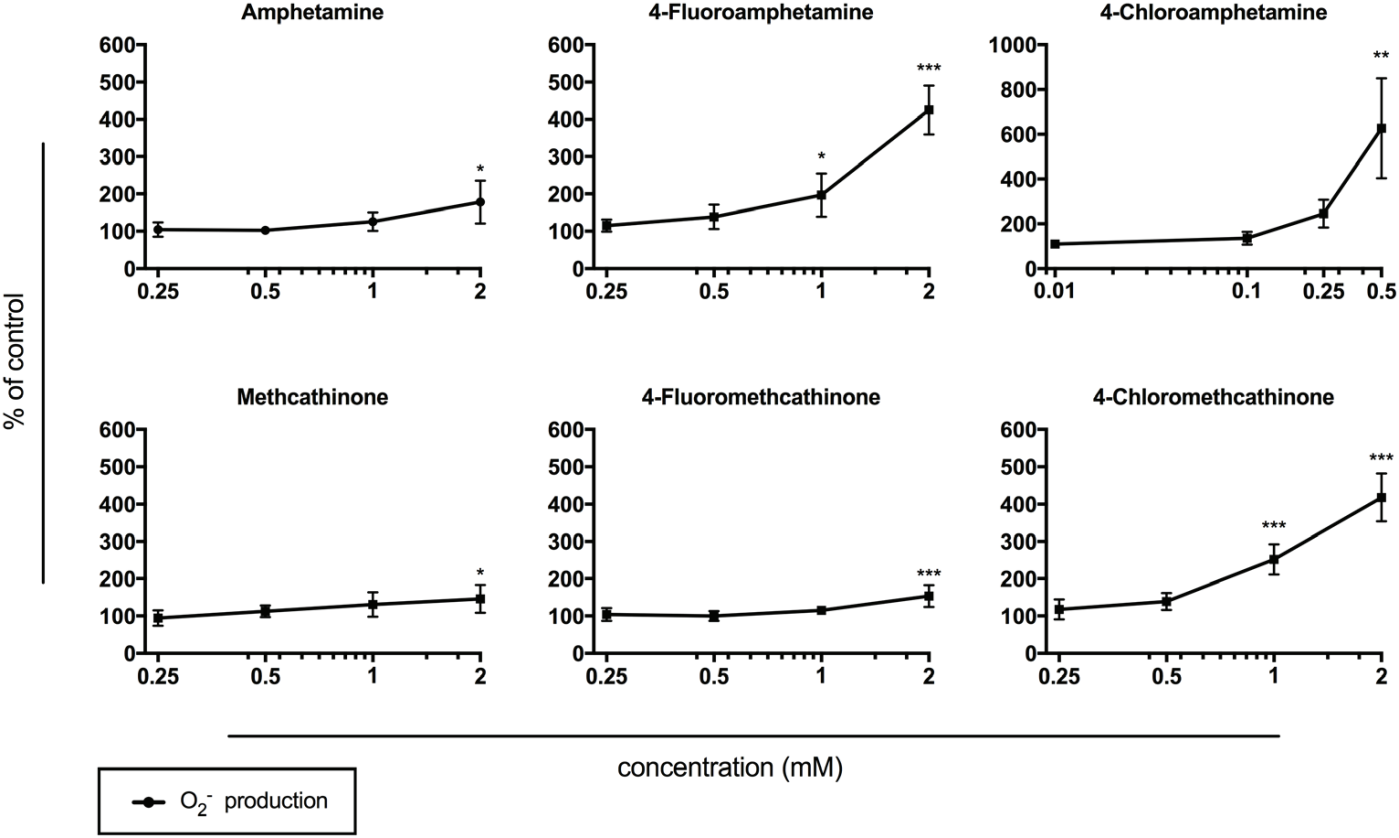
Mitochondrial superoxide production in HepG2 cells after drug exposure for 24 h. Data are expressed as mean ± SEM adjusted to protein content. Numbers in parentheses indicate the number of individual experiments performed in triplicate: amphetamine (5), 4-fluoroamphetamine (4), 4-chloroamphetamine (3), methcathinone (5), 4-fluoromethcathinone (4), and 4-chloromethcathinone (3). Significance levels are given as *p < 0.05, **p < 0.01, ***p < 0.001.

All test compounds induced a concentration-dependent increase in mitochondrial superoxide (Figure 5). A significant increase in mitochondrial superoxide was observed at 0.5 mM for 4-chloroamphetamine, at 1 mM for 4-fluoroamphetamine and 4-chloromethcathinone, and at 2 mM for amphetamine, methcathinone, and 4-fluoromethcathinone.

### Mechanisms of Cell Death

**Figure 6:**
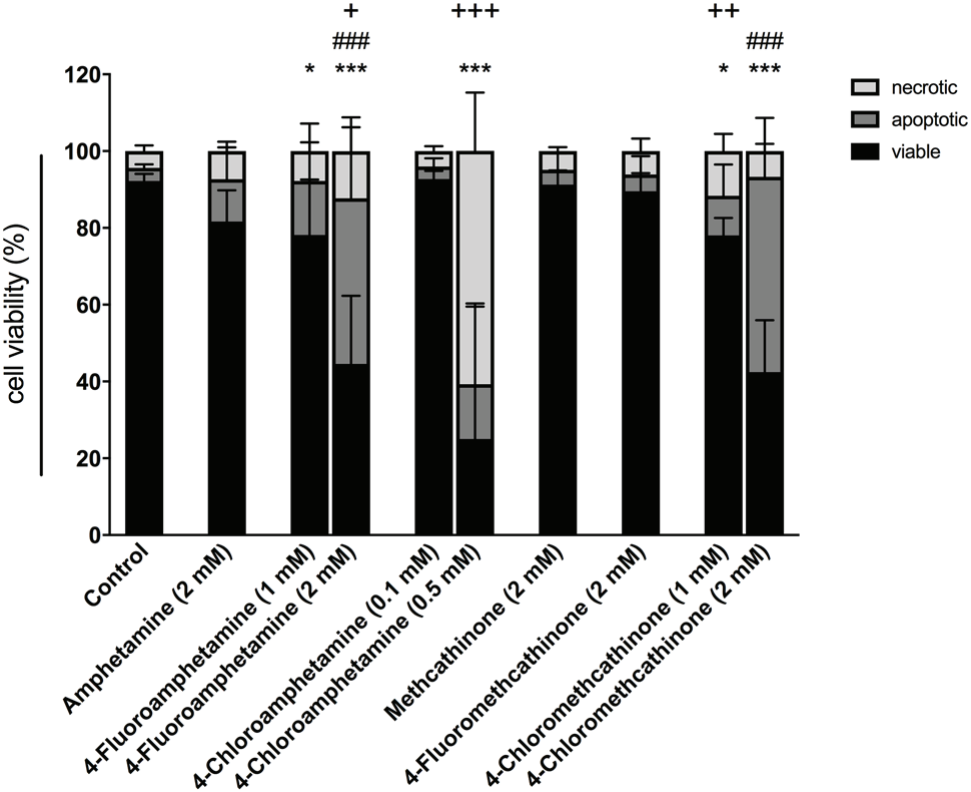
Mechanisms of cell death in HepG2 cells after drug exposure for 24 h. Data are expressed as mean ± SEM, and numbers in parentheses indicate the number of individual experiments: amphetamine (7), 4-fluoroamphetamine (8), 4-chloroamphetamine (4), methcathinone (7), 4-fluoromethcathinone (7), and 4-chloromethcathinone (7). Significance levels are given as *p < 0.05, ***p < 0.001 (viable cells); ###*p* < 0.001 (apoptotic cells); +*p* < 0.05, ++*p* < 0.01, +++*p* < 0.001 (necrotic cells).

The proportion of viable, apoptotic, and necrotic cells after treatment with the test drugs is shown in Figure 6. Using annexin V staining, a significant reduction in cell viability was observed for 4-fluoramphetamine and 4-chloromethcathinone starting at 1 mM and for 4-chloroamphetamine at 0.5 mM. Amphetamine, methcathinone, and 4-fluoromethcathinone did not decrease cell viability at 2 mM. Apoptosis was the main mechanism of death for HepG2 cells treated with 4-fluoroamphetamine and 4-chloromethcathinone, whereas the majority of cells treated with 0.5 mM 4-chloroamphetamine underwent necrosis.

## Discussion

### Pharmacological Perspective

*Para*-substituted amphetamines and methcathinones are monoamine transporter substrates (Rickli et al., 2015), elevating extracellular monoamine levels by inducing an efflux through reverse transport of the transporters (Rothman and Baumann, 2003; Sitte and Freissmuth, 2015). This subsequently results in inhibition of the monoamine uptake transport from the synaptic cleft.

Norepinephrine uptake inhibition was observed at submicromolar concentrations for all substances, and all compounds additionally inhibited the uptake of at least one other monoamine at low micromolar concentrations. As reported previously (Baumann et al., 2011; Simmler et al., 2014; Rickli et al., 2015; Suyama et al., 2016), *para*-halogenation reduced the selectivity for DAT over SERT, which is associated with a decreased abuse liability for amphetamine-type substances (Ritz et al., 1987; Kuhar et al., 1991; Baumann et al., 2000; Wee et al., 2005; Wee and Woolverton, 2006). *Para*-chlorination of amphetamine and methcathinone seems to reduce the selectivity for DAT over SERT in a similar manner as *para*-methylation (Luethi et al., 2018b). NET and DAT inhibition correlates with human effective doses, and SERT inhibition inversely correlates with human effective doses (Luethi and Liechti, 2018), indicating lower clinical potency for the halogenated amphetamine and methcathinone derivatives. However, the highly potent serotonergic activity coupled with considerably potent dopaminergic activity may explain the severe serotonergic neurotoxicity of 4-chloroamphetamine (Johnson et al., 1990). Currently, the neurotoxic potential of the corresponding methcathinone analog 4-chloromethcathinone is not well investigated.

### Toxicological Perspective

Amphetamine-type stimulant-induced organ damage is multifaceted and so far not completely understood (Carvalho et al., 2012). The amphetamines and methcathinones investigated in this study potently interacted with monoaminergic systems, which regulate body temperature in a variety of ways (Docherty and Green, 2010). Thus, hyperthermia is a possible consequence of the use of these substances, which can rarely lead to potentially fatal complications such as rhabdomyolysis, acute renal failure, acidosis, or multiple organ failure including the liver (Kendrick et al., 1977; Henry, 1992; Kalant, 2001). In addition to systemic causes of organ damage, the results of this study suggest that direct cellular mechanisms of halogenated amphetamines and methcathinones may contribute to liver and probably other organ toxicity. As observed in other studies (Dias da Silva et al., 2013, 2015; Luethi et al., 2017), HepG2 cells proved to be a robust cell model with low susceptibility to stimulant toxicity, allowing the investigation of cellular mechanisms of toxicity in the absence of cell death (Kamalian et al., 2015). The drug concentrations used were therefore mostly outside of the pharmacologically relevant range. However, concentrations inducing toxic effects may be lower in primary cells (Gerets et al., 2012), and the toxic effects of stimulants may be enhanced by elevated temperature (Valente et al., 2016). With limited diffusion across the cell membrane under physiological conditions, amphetamine and structurally related compounds require active transport to enter cells (Freyberg et al., 2016). However, as monoamine transporters are mainly expressed in the brain (Kristensen et al., 2011), amphetamine-type drug uptake into the liver likely relies on other transporters. A previous study of the gene expression for various transporters revealed substantially decreased overall transporter expression in HepG2 cells compared to human liver tissue (Hilgendorf et al., 2007). This may be one of the reasons why these cells are more resistant to amphetamine-type drug toxicity. Therefore, reduced transporter expression in HepG2 cells may be one compared to primary cells. Therefore, even though this study provides information on possible cellular mechanisms that could contribute to clinical toxicity in stimulant users, it does not provide precise information about the toxic concentration range. Furthermore, organ-toxic drug levels and mechanisms of toxicity may vary considerably between users. For example, case reports of liver damage associated with the amphetamine derivative 3,4-methylenedioxymethamphetamine (MDMA) do not indicate strictly dose-dependent hepatotoxicity (Ellis et al., 1996). The present study therefore demonstrates underlying cellular mechanisms that may contribute to clinical toxicity in susceptible drug users. Both *para*-fluorination and *para*-chlorination increased the cytotoxicity of amphetamine and methcathinone, with a higher toxicity observed for amphetamines compared to the corresponding methcathinones. For all substances, a decrease in cellular ATP preceded the loss in cell membrane integrity, which points out the possibility of mitochondrial impairment. A more detailed investigation of the mitochondrial respiration revealed that the investigated amphetamines in fact interfered with mitochondrial function by decreasing basal respiration. In addition, both halogenated amphetamine derivatives additionally decreased the maximal respiratory capacity of HepG2 cells. While methcathinone and 4-fluoromethcathinone did not significantly alter cellular respiration, a decrease in the maximal respiration was observed for 4-chloromethcathinone. Furthermore, 4-chloromethcathinone was the only compound to uncouple cellular respiration from ATP synthesis. Such uncoupling properties have previously been observed for the structurally similar fluorinated cathinone derivative bupropion (Luethi et al., 2017). Disruption of the mitochondrial respiratory chain is associated with increased ROS levels, with the enzyme complex I and III as potential sites for ROS generation (Votyakova and Reynolds, 2001; Antico Arciuch et al., 2012; Dröse and Brandt, 2012). Increased ROS levels may subsequently elicit toxicity by causing damage to various cellular components. A concentration-dependent increase in ROS was observed in HepG2 cells after treatment with all investigated compounds, which may be explained by the disruption of the mitochondrial electron transport chain. Additionally, impairment of the cellular antioxidant response could contribute to increased ROS levels; this was, however, not investigated in this study. Investigations of cell death revealed a concentration-dependent increase in apoptotic cells after treatment with 4-fluoroamphetamine and 4-chloromethcathinone; a concentration-dependent increase in necrotic cells was observed after treatment with 4-chloroamphetamine. These observations are consistent with the notion that ATP levels are a determinant of manifestation of cell death (Eguchi et al., 1997). As shown in this study, a decrease in ATP after 4-chloroamphetamine treatment is much more distinct when compared to the other compounds. At 0.5 mM, treatment with 4-chloroamphetamine caused an almost complete ATP depletion, explaining the high amount of necrotic cells at this concentration. The amount of necrotic and apoptotic cells after treatment with 2 mM amphetamine, methcathinone, and 4-fluoromethcathinone did not significantly differ from control incubations. As no concentrations higher than 2 mM were investigated in this study, no predictions about the amount of necrotic and apoptotic cells at higher concentrations or about dose-dependency can be made.

The investigations of this study were restricted to *para*-halogenated derivatives of amphetamine and methcathinone. Whether *ortho*- or *meta*-halogenation results similarly in increased hepatocellular toxicity remains to be investigated in a future study.

## Conclusion

All investigated compounds inhibited norepinephrine uptake at submicromolar concentrations and dopamine uptake at low micromolar concentrations. In addition, *para*-fluorination and *para*-chlorination increased the potential to inhibit serotonin uptake, which could result in severe serotonergic neurotoxicity. In comparison to the non-halogenated compounds, *para*-halogenation of amphetamine and methcathinone furthermore increased the cytotoxicity of the respective derivatives in HepG2 cells. The results of this study suggest mitochondrial toxicity for amphetamine and its halogenated derivatives as well as for 4-chloromethcathinone. Methcathinone and 4-fluoromethcathinone may potentially disrupt mitochondrial function as well; however, this was not observed at investigated concentrations in this study (≤2 mM). The potent monoamine transporter inhibition potential of the substances indicates that adverse effects are most likely linked to sympathomimetic toxicity, which is consistent with clinical intoxication reports (Knippels et al., 2017; Wijers et al., 2017; Hondebrink et al., 2018). Direct cellular mechanisms, such as mitochondrial toxicity, may, however, additionally play a contributory role in susceptible users.

## Author Contributions

DL, SK, and ML designed the research. DL, MW, XZ, and DR conducted the research. DL, DR, SK, and ML analyzed the data. DL, SK, and ML wrote the manuscript with significant input from all the other authors.

### Conflict of Interest Statement

The authors declare that the research was conducted in the absence of any commercial or financial relationships that could be construed as a potential conflict of interest.
